# Supplementary material for: Lysosomal SLC46A3 modulates hepatic cytosolic copper homeostasis
Source: Nat Commun. 2021 Jan 12;12:290. doi: 10.1038/s41467-020-20461-0 (PMC7804329; doi:10.1038/s41467-020-20461-0)
Supplement: Supplementary file 1 — Supplementary Information [file 41467_2020_20461_MOESM1_ESM.pdf]

Supplementary Information

Lysosomal SLC46A3 modulates hepatic cytosolic copper homeostasis
Kim, J et al.,

Methods

Lipidomics by LC-MS and multivariate data analysis

Fifty milligrams of liver tissue from 22- to 25-week-old male mice were mixed with 300 μl of water and 400 μl of methanol containing 5 μM of chloropropamide and homogenized with Precellys 24 homogenizer (Bertin Instrumnets, MD). The tissue suspensions (375 μl) were mixed with 400 μl of chloroform containing phosphatidylcholine (PC,17:02, 5 μM) followed by shaking incubation at 37°C for 20 min. The samples were centrifuged at 18,000g for 20 min at 4°C and the organic phases carefully collected, evaporated under nitrogen flow and dissolved with 100 μL of methanol/chloroform (1:1). After 50-fold dilution with injection buffer (isopropanol: acetonitrile: H_2O=2:1:1), the samples were subjected to UPLC-ESI-QTOFMS as described previously[1]. Centroided and integrated chromatographic mass data were processed by MarkerLynx (Waters) to generate a multivariate data matrix. Pareto-scaled MarkerLynx matrices including information on sample identity were analyzed by Partial least squares-discriminant analysis (PLS-DA) analysis using SIMCA-P+12 (Umetrics, Kinnelon, NJ). The PLS-DA loading scatter S-plot was used to determine the lipids that contributed significantly to the separation between WT and *Slc46a*$^{-/-}$ mice. The lipid metabolite structures were determined using the METLIN metabolite database and gas chromatography after fatty acid FAME-derivatization. The identity of TG species in the liver was confirmed by tandem mass spectrometry MS/MS fragmentation patterns. Chlorpropamide and PC (17:02) were used as internal standards.

Supplementary Table 1. Sequences of primers used in PCR analysis

a) qPCR primers

Gene	Forward (5' to 3')	Reverse (5' to 3')
Gapdh	CGTCCCGTAGACAAAATGGT	TTGATGGCAACAATCTCCAC
18S	ATTGGAGCTGGAATTACCGC	CGGCTACCACATCCAAGGAA
Cyp1a2	GGAGAATGTCACCTCAGGGAAT	CGAAGTTATCATTGAAGGTCTTAAAC
Slc46a3	GGAGGAAGTTCAGAAAAAGGCATC	TGTCGCTGCTCGCTAAAAGC
Acadm	AGCTCTAGACGAAGCCACGA	GCGAGCAGAAATGAAACTCC
Acox1	TCGCAGACCCTGAAGAAATC	CCTGATTCAGCAAGGTAGGG
Fasn	AAGTTGCCCGAGTCAGAGAACC	ATCCATAGAGCCCAGCCTTCCATC
Acc1	TGGTGCAGAGGTACCGAAGTG	CGTAGTGGCCGTTCTGAAACT
Scd1	ACGCCGACCCTCACAATTC	CAGTTTTCCGCCCTTCTCTTT
Lcat	GGTTTTATCTCTCTCGGGGC	TATGTTGGACAGGATGGGGA
Cd36	GCGACATGATTAATGGCACA	CCTGCAAATGTCAGAGGAAA
Hsl	CCTCCAAGCAGGGCAAAGA	GCGTAAATCCATGCTGTGTGA
Pnpla2	CCACTCACATCTACGGAGCC	TAATGTTGGCACCTGCTTCA
Aadac	ACCGCTTCCAGATGCTATTG	TGATTCCCAAAAGTTCACCA
Tgh	TGGTATTTGGTGTCCCATCA	GCTTGGGCGATACTCAAACT
Mttp	CGTGGTGAAAGGGCTTATTC	TCGCGATACCACAGAATGAA
Dgat1	GACGGCTACTGGGATCTGA	TCACCACACACCAATTCAGG
Dgat2	CGCAGCGAAAACAAGAATAA	GAAGATGTCTTGGAGGGCTG
Fsp27	TGGGAGGTCCAACACAATCCA	GTGCTCACTGCCACATGCCT
Adfp	GCTCTCCTGTTAGGCGTCTC	GTAGAGCTCACCAAGGGCAG
Trf	TGTCTGGCTGTCCCTGACAA	CTTCATGTGGTCACGGAAGCT
Tfrc	ACACCCGGTTTAGCCTTGCT	TGTCGGCATTTTCTTCTTCATCT
Fth1	CAGCGAGGTGGCCGAAT	CAGTGCACACTCCATTGCATT
Ftl1	AGCGTCTCCTCGAGTTTCAGAA	GGGTTTTACCCCATTCATCTTG
Zip5	CAGCTGCTGGTCAATTTTGGA	CACGGCTGTCAATCTGATAAAGC
Zip14	ATGCGTCTGCAACTCGTGACT	CACCACCTGCCACGTCACT
Znt9	CTAGGTGTGGTCTCGGCATTC	AGTAAGCCGCTGCACTTGCT
ZnT7	CCCCTGTCCATCAAGGATGA	GTCCGACAGGATGGACCTAAAC
Slc26a3	GGTTGGGAACATGAGTCTTGGA	GCCGAAGCTGTCTCCTATGG
Atp7a	GGATCACCACCTTGCAACTCTT	TGACGCTCCAGAAACATAGCA
Atp7b	GATTGGAAACCGGGAATGG	GATGGCCGTCTGTCCTTTCA
Ahr	AGCCGGTGCAGAAAACAGTAA	AGGCGGTCTAACTCTGTGTTC

b) PCR primers for Southern Blotting Probe

Mouse Slc46a3	Sequence (5' to 3')
Forward	ATTGCCCCTCCACTGTAAGACCC
Reverse	GATTTGTGAAGTAACCGCTCCTCTCC

Supplementary Figures

Supplementary Figure 1. Hepatic metal profiles and their related gene expressions.

Supplementary Figure 2. Isolation of lysosome using eGFP-LAMP1.

Supplementary Figure 3. Ablation of the *Slc46a3* gene does not alter the clearance of blood glucose.

Supplementary Figure 4. Ablation of the *Slc46a3* gene decreases hepatic triglyceride level.

Supplementary Figure 5. Oil Red O staining of Hepa1c1c7 cells after copper chelator treatment.

Supplementary Figure 6. Analysis of 63Cu and 56Fe in eGFP-SLC46A3 expressing liver.

Supplementary Figure 7. TCDD increases glycosylated Lamp1 in mouse liver.

Supplementary Figure 8. Copper staining with rhodanine in the presence or absence of SLC46A3 in mouse liver.

Supplementary Figure 9. Mitochondrial fission or fusion is not affected by SLC46A3.

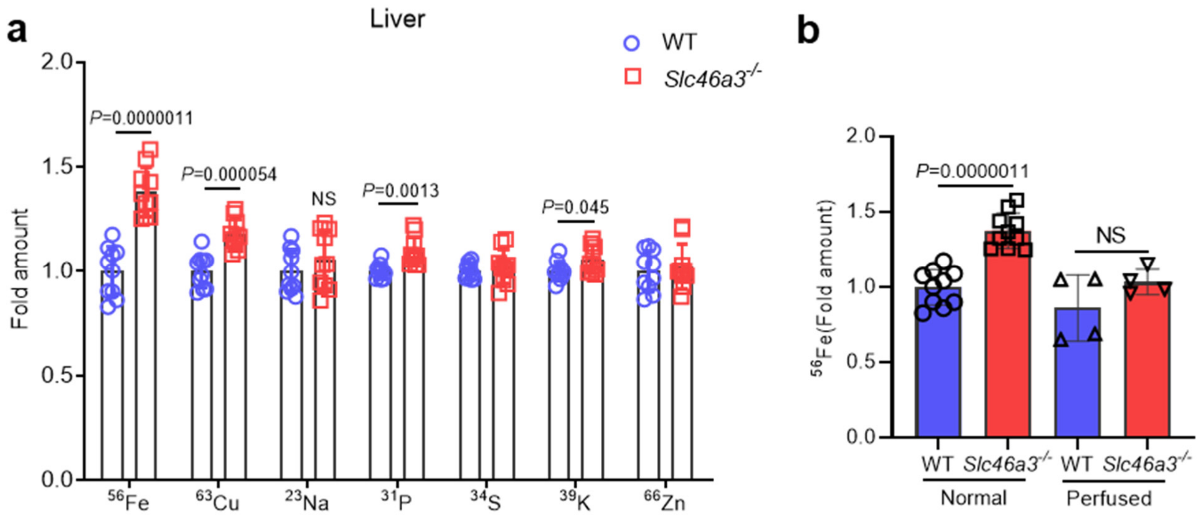

Supplementary Figure 1. Hepatic metal profiles and their related gene expressions. a. Inductively coupled plasma mass spectrometry shows different species of metals in liver. **b**. Iron (^{56}Fe) was measured before and after liver perfusion with phosphate buffered-saline. Each data point represents the mean ± SD and adjusted *p* value, presented in the panels, was determined by unpaired two-tailed Student's *t* test using indicated sample sizes and groups. NS, not significant.

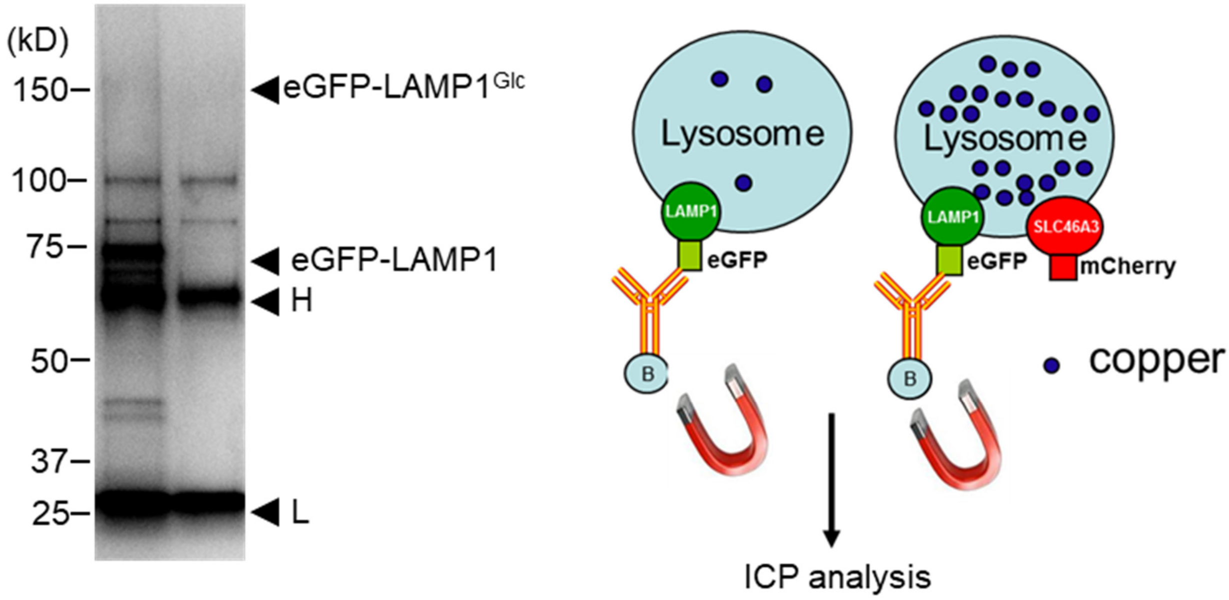

Supplementary Figure 2. Isolation of lysosome using eGFP-LAMP1. To isolate lysosomes from cells, lysates were obtained using a mitochondrial isolation kit (Thermo). Then, lysates were incubated with GFP antibody to pull down the eGFP-LAMP1 or LAMP1-eGFP. Western blot data shows isolated eGFP-LAMP1 protein. The representative western blot image was obtained from three independent experiments. Glc, glycosylated; H, heavy chain; L, light chain.

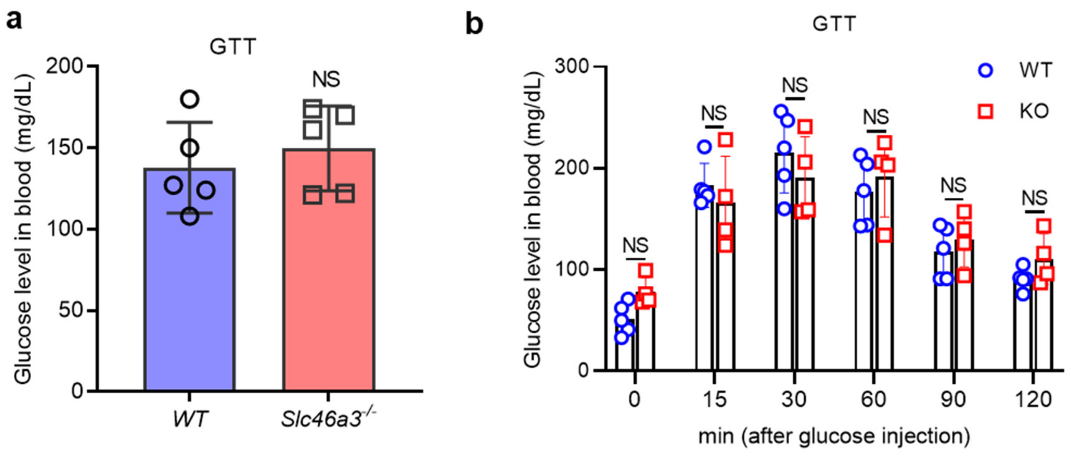

Supplementary Figure 3. Ablation of the *Slc46a3* gene does not alter the clearance of blood glucose. a. Eight-week old WT (n=5 mice per group) and *Slc46a3*$^{-/-}$ (n=5 mice per group) mice were subjected to a glucose tolerance test (GTT). **b**. Fifteen-week-old WT (n=5 mice per group) and *Slc46a3*$^{-/-}$ (n=4 mice per group) mice were subjected to a glucose tolerance test (GTT)[2]. Each data point represents the mean ± SD; NS, not significant.

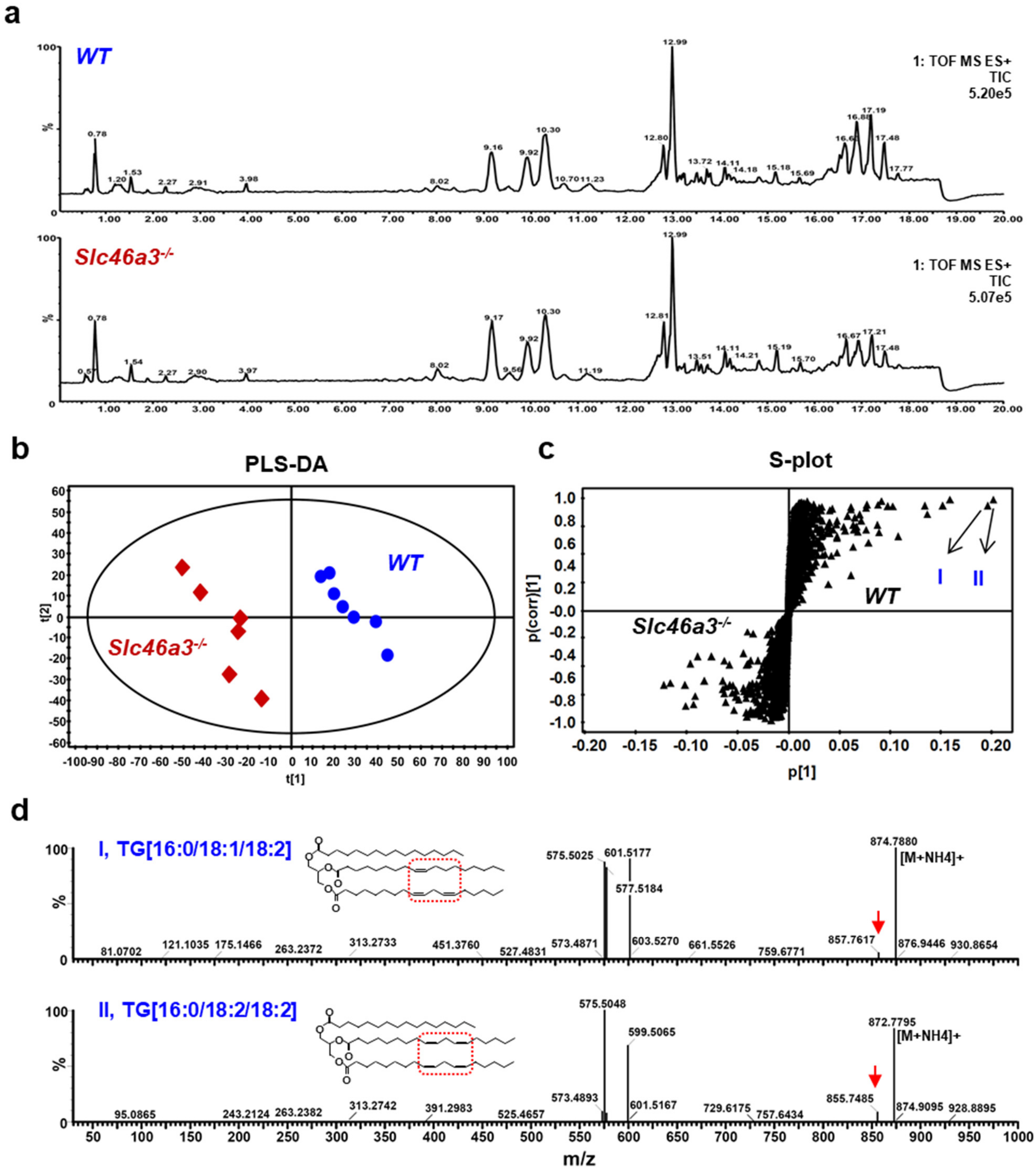

Supplementary Figure 4. Ablation of the *Slc46a3* gene decreases hepatic triglyceride levels. **a.** Representative images of the UPLC chromatogram of hepatic lipid extracts from WT and *Slc46a3*$^{-/-}$ mice. n=6-7 mice/group. **b.** Partial least squares-discriminant analysis (PLS-DA) of (**a**). **c.** S-plot analysis from (**a**). Two abundant ions from WT are indicated as I and II. **d.** UPLC-MS profile shows the possible TG ions (I and II). Red arrows, actual mass.

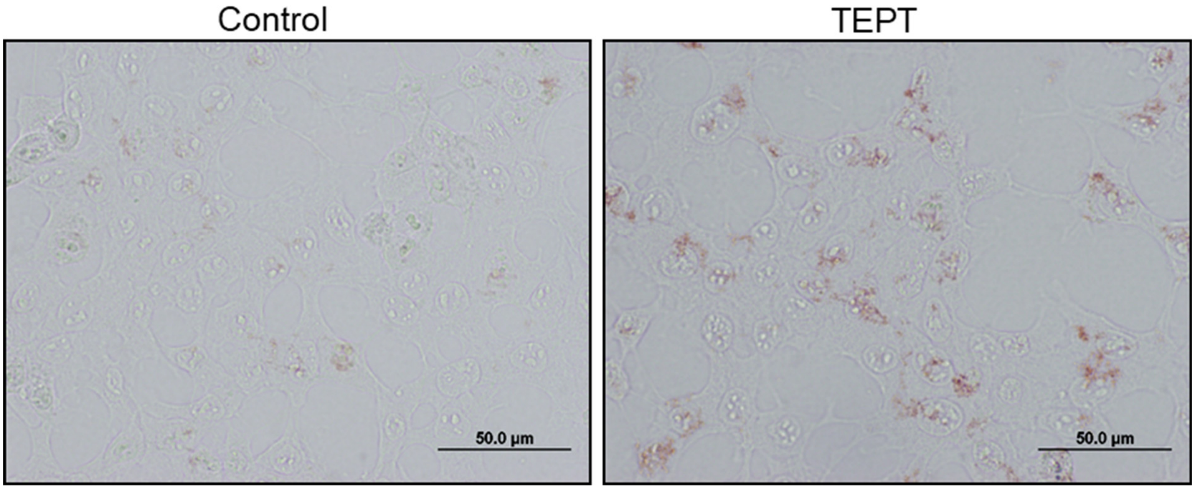

Supplementary Figure 5. Oil Red O staining of Hepa1c1c7 cells after copper chelator treatment.

The cells were treated with 50 μM tetraethylenepentamine (TEPT) for 20 h and subjected to Oil Red O staining. The representative image was obtained from three independent experiments.

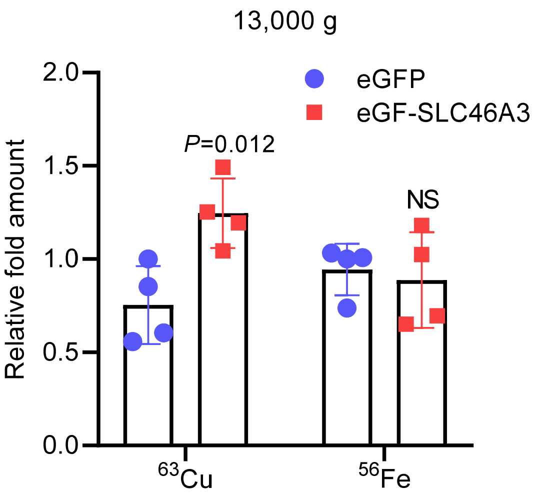

Supplementary Figure 6. Analysis of ^{63}Cu and ^{56}Fe in eGFP-SLC46A3 expressing liver. Inductively coupled plasma mass spectrometry (ICP) analysis of ^{63}Cu and ^{56}Fe with pellet of 13,000 g in eGFP (n=4 biologically independent experiments) or eGFP-SLC46A3 (n=4 biologically independent experiments) expressing liver. NS, not-significant. Each data point represents the mean ± SD and adjusted *p* value, presented in the panels, was determined by unpaired two-tailed Student's *t* test using indicated sample sizes and groups.

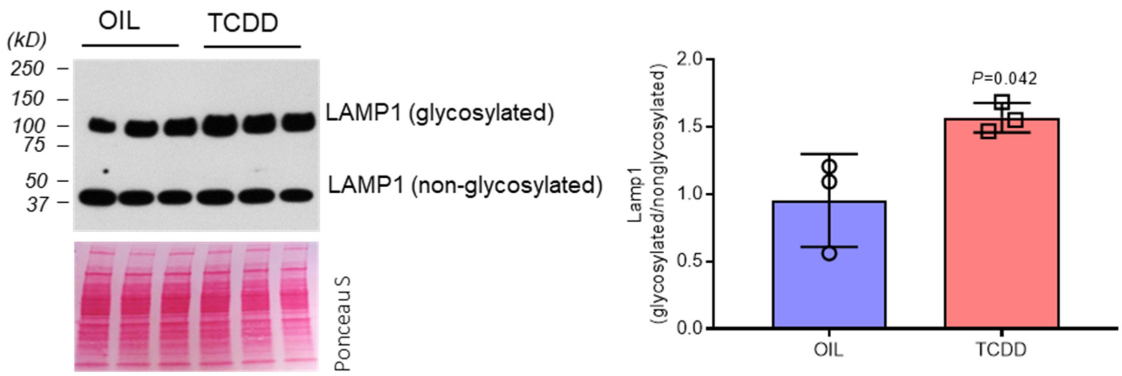

Supplementary Figure 7. TCDD increases glycosylated Lamp1 in mouse liver. Western blot data show the increase of hepatic glycosylated Lamp1 protein after TCDD (400 ng/kg for 7 day) treatment. The representative image was obtained from three independent experiments. Densitometrical measurement is shown in the right panel. Each data point represents the mean ± SD and adjusted *p* value, presented in the panels, was determined by unpaired two-tailed Student's *t* test using indicated sample sizes and groups.

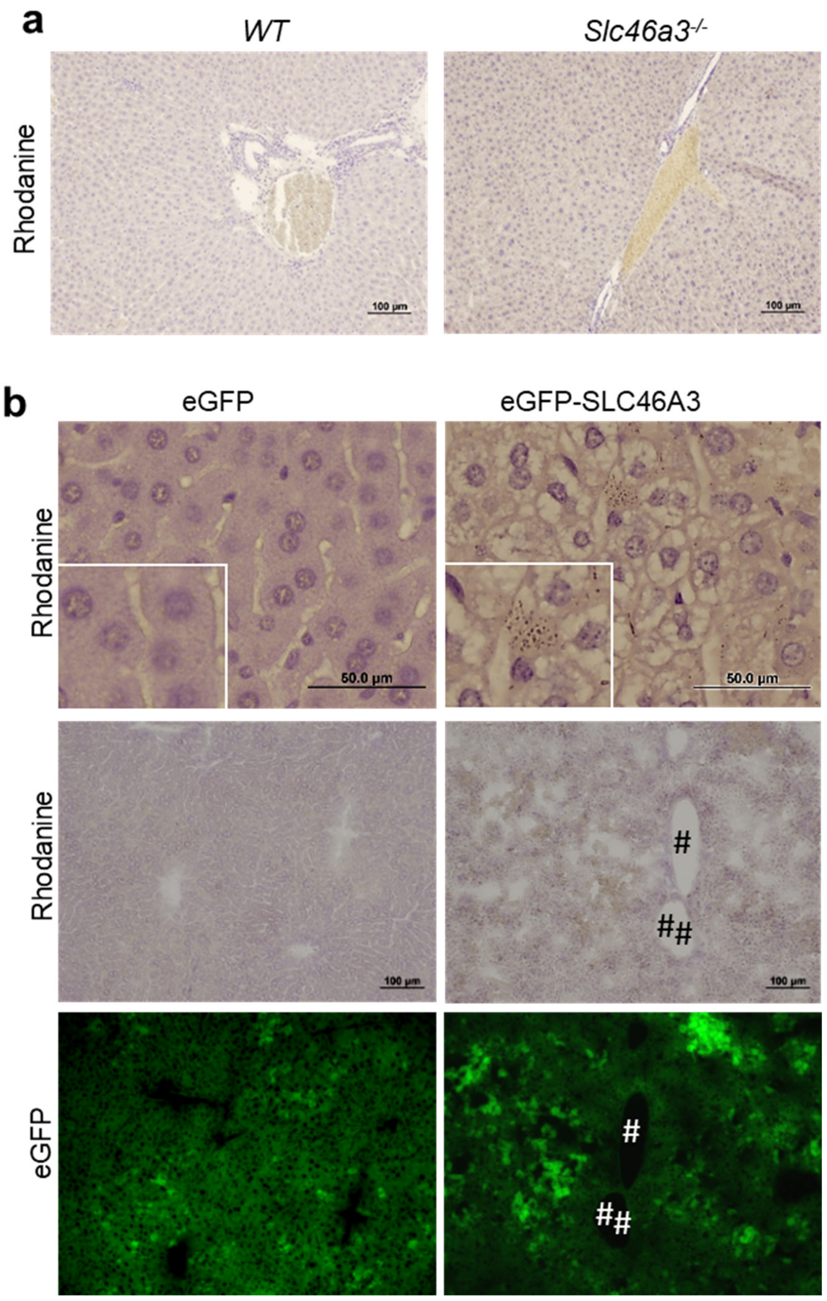

Supplementary Figure 8. Copper staining with rhodanine in the presence or absence of SLC46A3 in mouse liver. **a**. Copper staining with rhodanine using liver samples from WT and *Slc46a3*$^{-/-}$ mice. **b**. Copper staining with rhodanine using eGFP or eGFP-SLC46A3 expressed in liver. # and ## denote the same position in successive cuts of the paraffin sample.

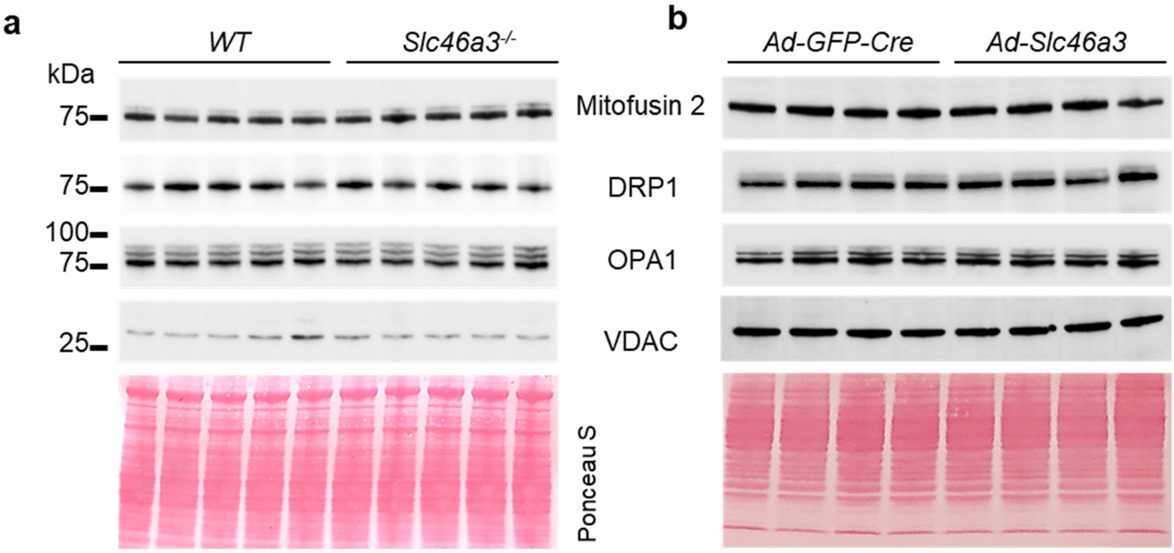

Supplementary Figure 9. Mitochondrial fission or fusion is not affected by SLC46A3. Liver samples from *Slc46a3*$^{-/-}$ mice (n=5 biologically independent liver samples) (**a**) and adenoviral SLC46A3 expressing mice (n=4 biologically independent liver samples) (**b**) were subjected to western blotting. Antibodies against mitochondrial fission or fusion were used.

References

1. Tanaka N*, et al.* Growth arrest and DNA damage-inducible 45alpha protects against nonalcoholic steatohepatitis induced by methionine- and choline-deficient diet. *Biochim Biophys Acta Mol Basis Dis* **1863**, 3170-3182 (2017).

2. Tanaka N*, et al.* Adipocyte-specific disruption of fat-specific protein 27 causes hepatosteatosis and insulin resistance in high-fat diet-fed mice. *J Biol Chem* **290**, 3092-3105 (2015).
