# Supplementary material for: Lysosomal SLC46A3 modulates hepatic cytosolic copper homeostasis
Source: Nat Commun. 2021 Jan 12;12:290. doi: 10.1038/s41467-020-20461-0 (PMC7804329; doi:10.1038/s41467-020-20461-0)
Supplement: Supplementary file 3 — Source Data [file 41467_2020_20461_MOESM3_ESM.zip › SOURCE~1.PDF]

Source Data

Full panels of western blots in figures and supplementary figures

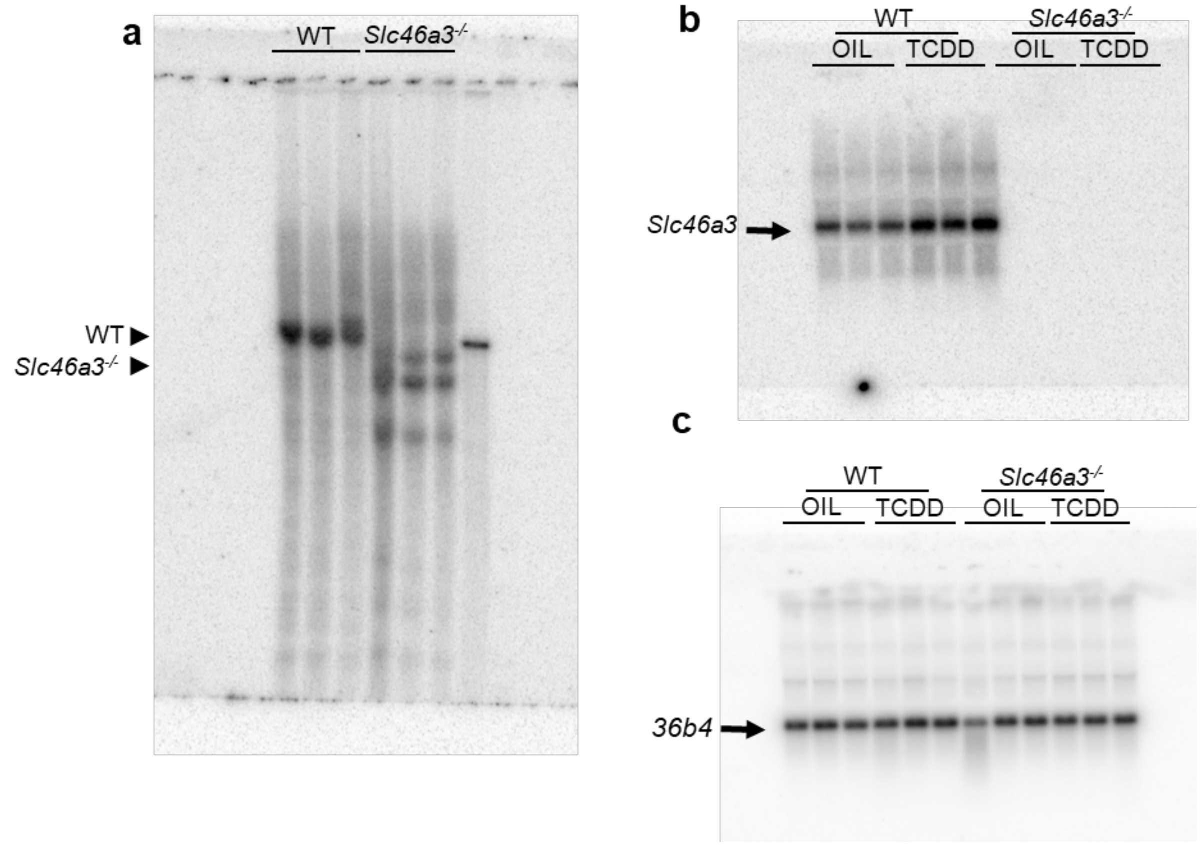

Related to Figure 2b and 2c. Full Southern and full Northern panels. a. WT and *Slc46a3*$^{-/-}$ from which the data in Figure 2b was derived. **b.** *Slc46a3* from which the data in Figure 2c (upper) was derived. **c.** *36b4* from which the data in Figure 2c (lower) was derived.

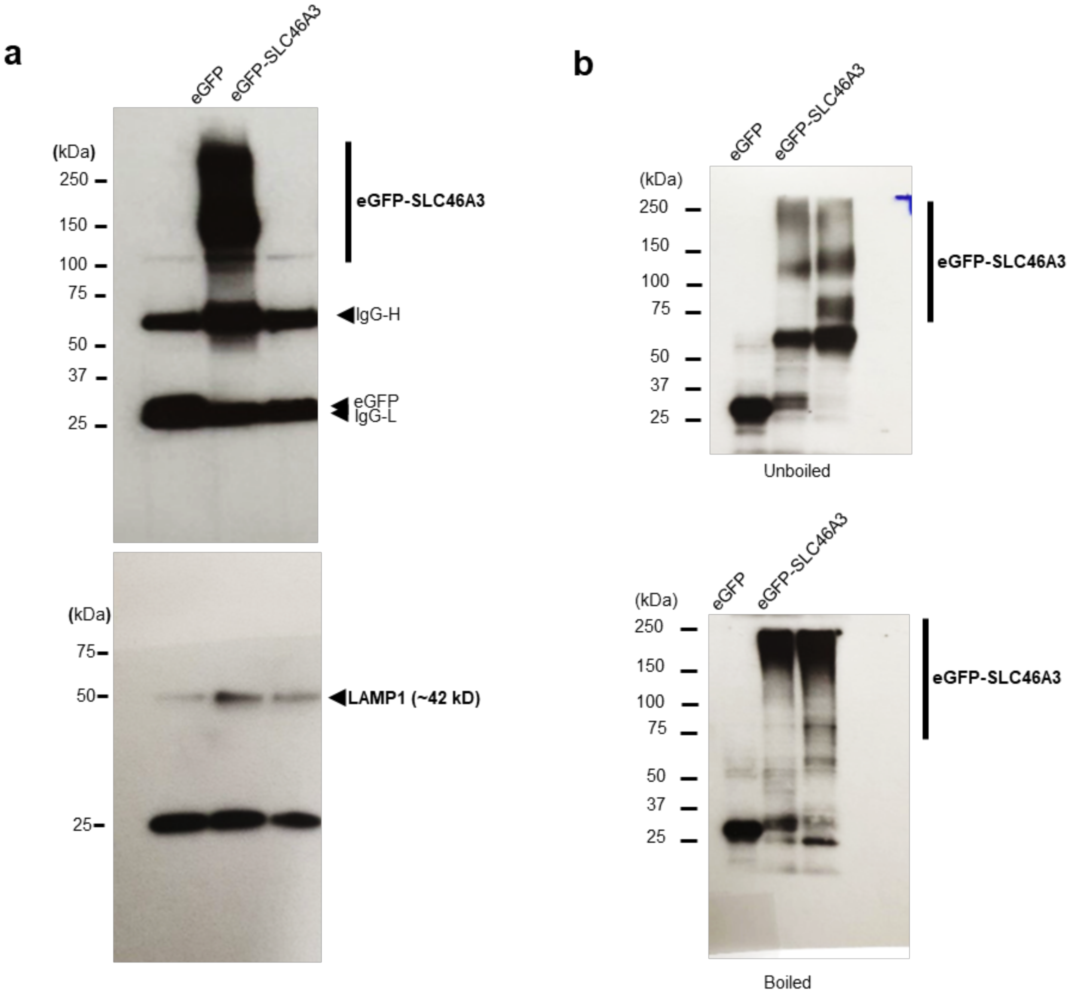

Related to Figure 2g and 2h. Full western blot panels. a. eGFP-SLC46A3 and LAMP1 (glycosylated) from which the data in Figure 2g was derived. **b.** eGFP-SLC46A3 from which the data in Figure 2h was derived.

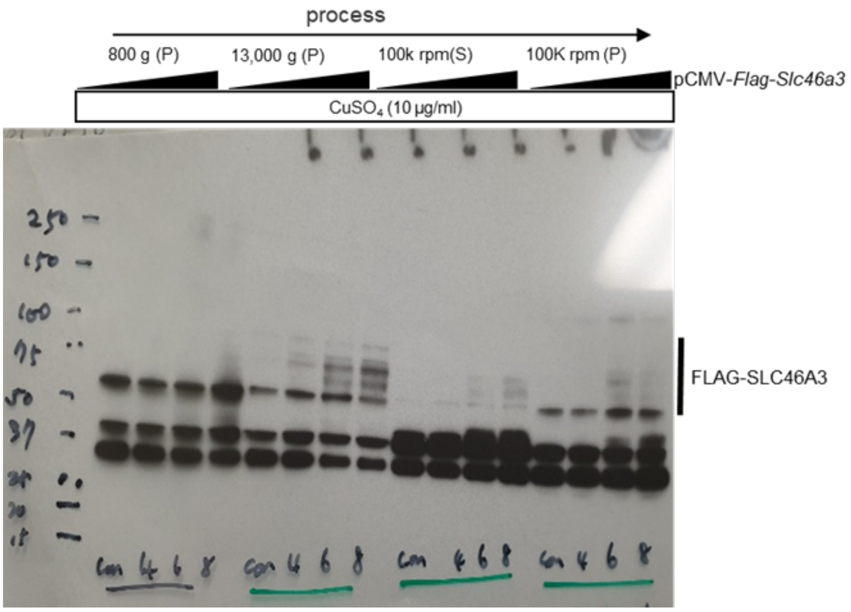

Related to Figure 3c. Full western blot panels. FLAG-SLC46A3 from which the data in Figure 3c was derived.

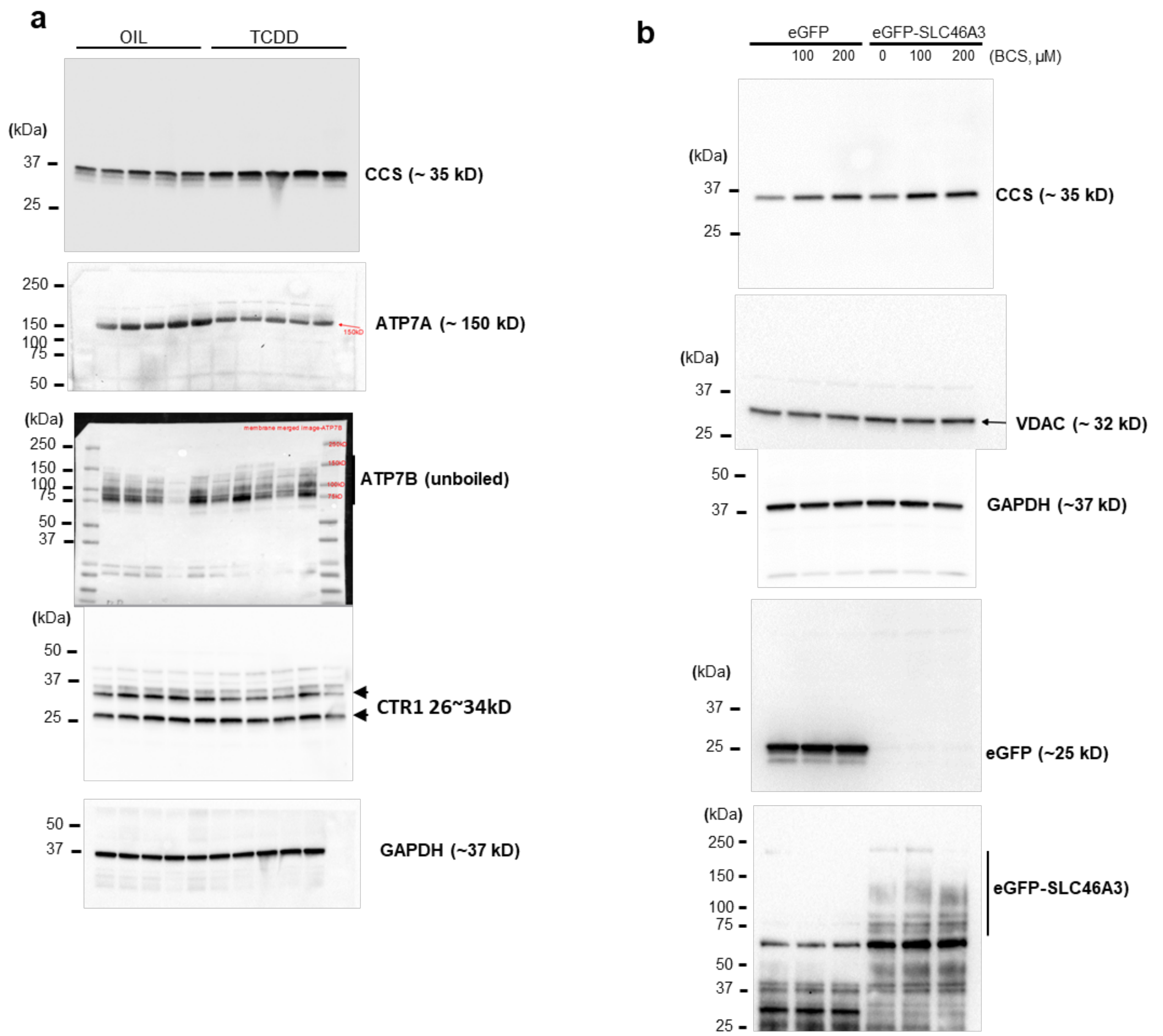

Supplementary Figure 4a and 4b. Full western blot panels. a. CCS, ATP7A, ATP7B, CTR1 and GAPDH from which the data in Figure 4a was derived. **b.** CCS, VDAC, GAPDH, eGFP and eGFP-SLC46A3 from which the data in Figure 4b was derived.

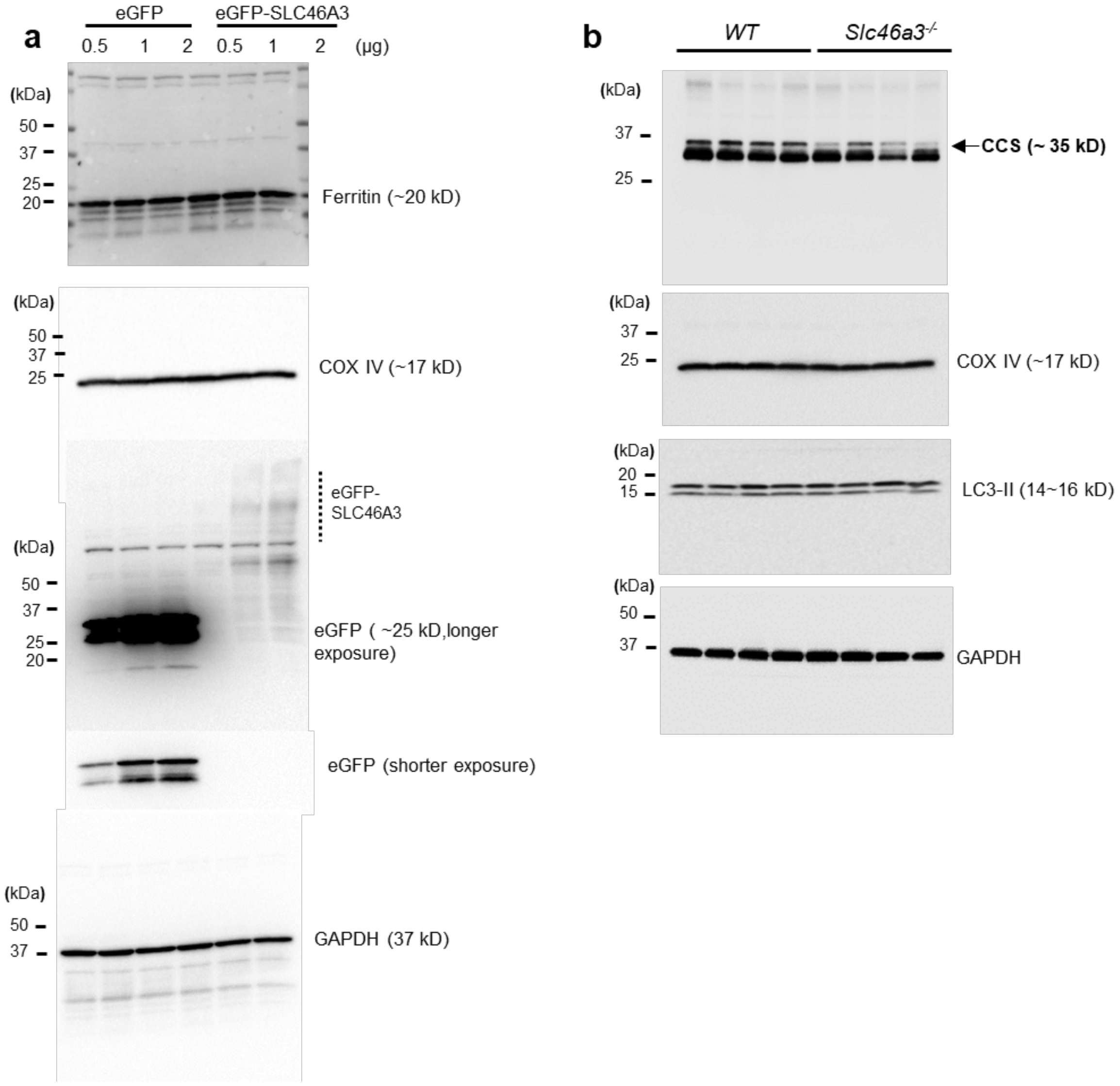

Related to Figure 4c. a. Ferritin, COX IV, eGFP-SLC46A3, eGFP and GAPDH from which the data in Figure 4c was derived. **b.** CCS, COX IV, LC3-II and GAPDH from which the data in Figure 4e was derived.

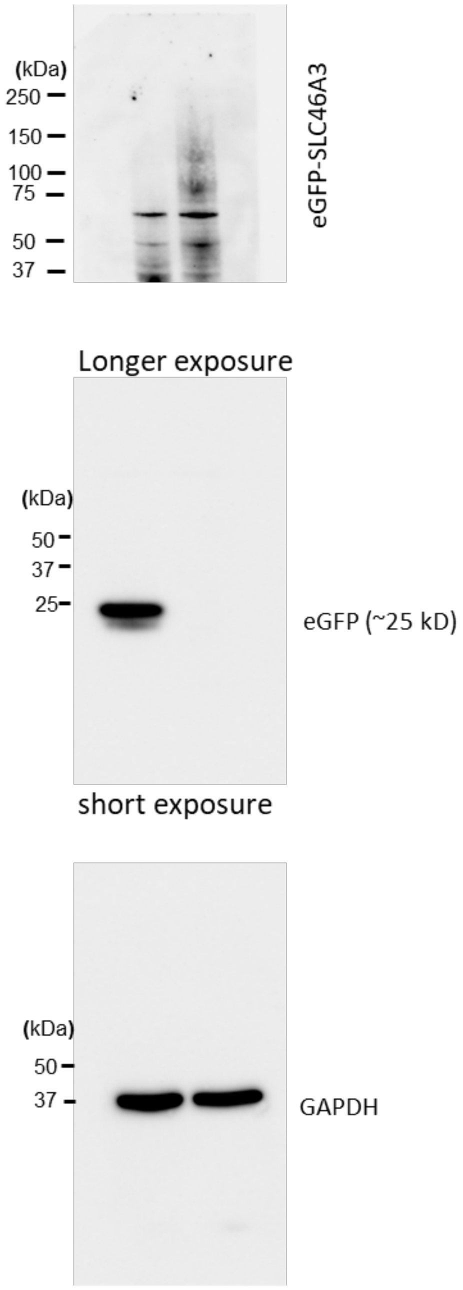

Related to Figure 5g. Full western blot panels. eGFP-SLC46A3 and eGFP and GAPDH from which the data in Figure 5g was derived.

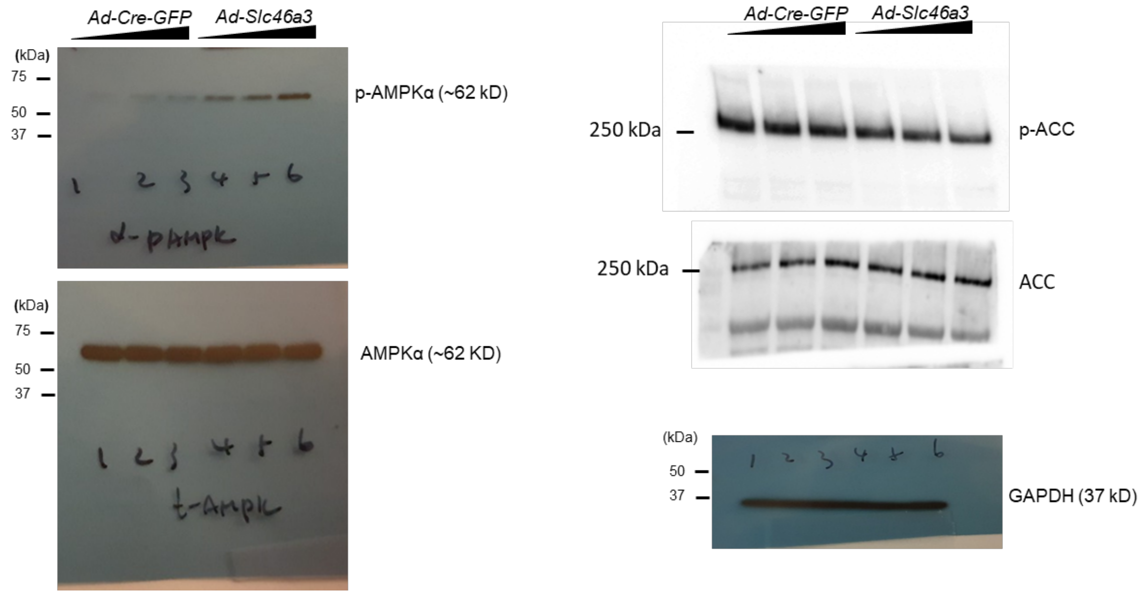

Related to Figure 7g. p-AMPK, AMPK, p-ACC, ACC and GAPDH from which the data in Figure 7g was derived.

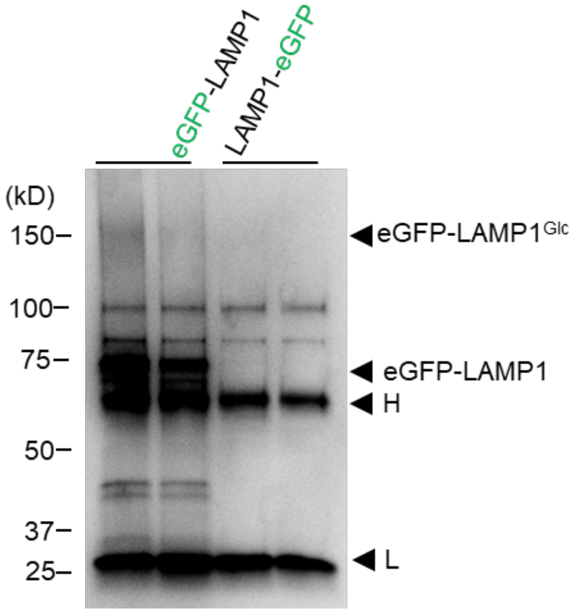

Related to Figure S2. Full western blot panels. eGFP-LAMP1 from which the data in Figure S2 was derived.

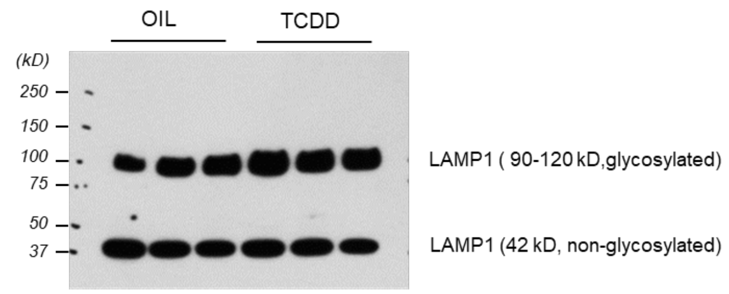

Related to Figure S7. Full western blot panels. LAMP1 from which the data in Figure S7 was derived.

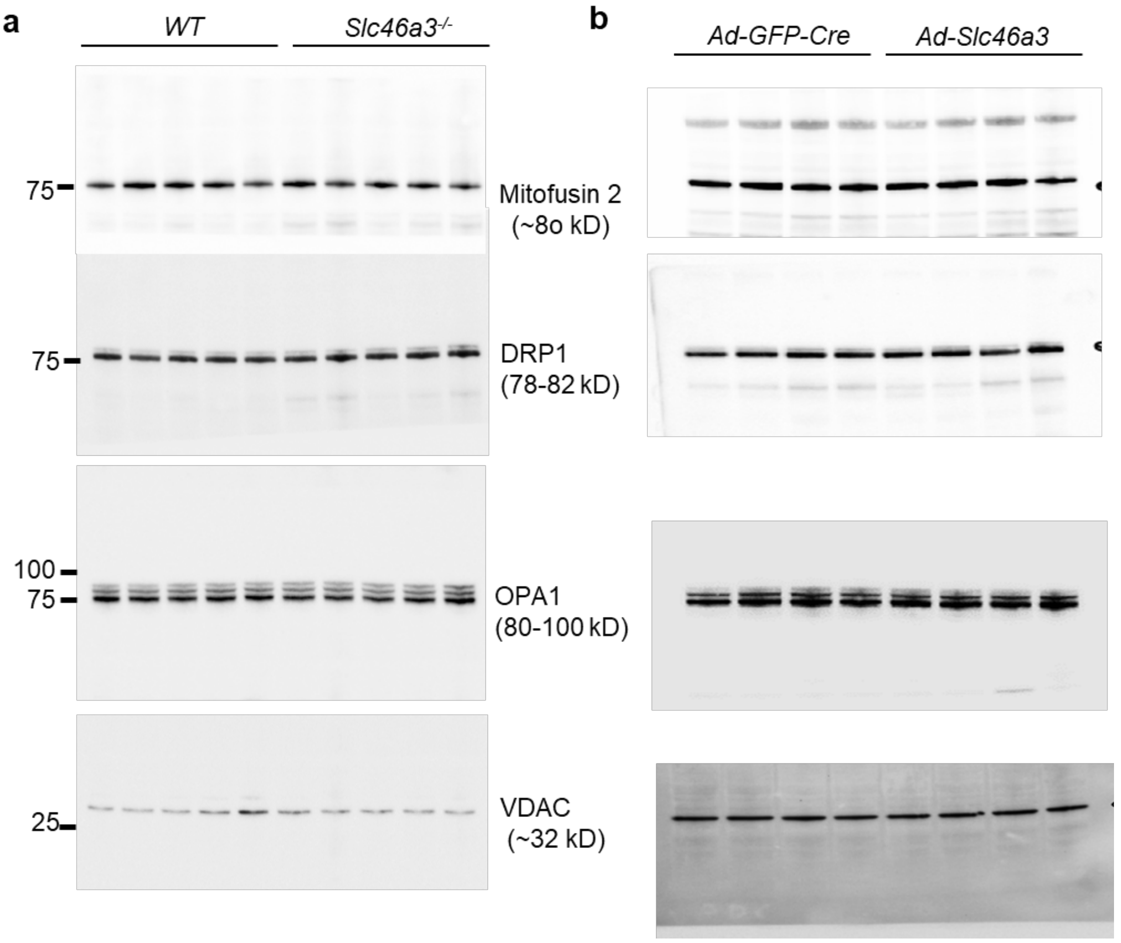

Related to Figure S9b. a. Mitofusin 2, Drp1, OPA1 and VDAC from which the data in Figure S9a was derived. **b.** Mitofusin 2, Drp1, OPA1 and VDAC from which the data in Figure S9b was derived.
